# Electrochemical Acetylcholinesterase Sensors for Anti-Alzheimer’s Disease Drug Determination

**DOI:** 10.3390/bios14020093

**Published:** 2024-02-09

**Authors:** Alexey Ivanov, Rezeda Shamagsumova, Marina Larina, Gennady Evtugyn

**Affiliations:** 1 A.M. Butlerov’ Chemistry Institute, Kazan Federal University, 18 Kremlevskaya Street, 420008 Kazan, Russia; rezeda.shamagsumova@kpfu.ru (R.S.); gennady.evtugyn@kpfu.ru (G.E.); 2 Institute of Fundamental Medicine and Biology, Kazan Federal University, 18 Kremlevskaya Street, 420008 Kazan, Russia; mllarina@kpfu.ru; 3 Analytical Chemistry Department, Chemical Technology Institute, Ural Federal University, 19 Mira Street, 620002 Ekaterinburg, Russia

**Keywords:** electrochemical biosensor, acetylcholinesterase, reversible inhibition, drug determination

## Abstract

Neurodegenerative diseases and Alzheimer’s disease (AD), as one of the most common causes of dementia, result in progressive losses of cholinergic neurons and a reduction in the presynaptic markers of the cholinergic system. These consequences can be compensated by the inhibition of acetylcholinesterase (AChE) followed by a decrease in the rate of acetylcholine hydrolysis. For this reason, anticholinesterase drugs with reversible inhibition effects are applied for the administration of neurodegenerative diseases. Their overdosage, variation in efficiency and recommendation of an individual daily dose require simple and reliable measurement devices capable of the assessment of the drug concentration in biological fluids and medications. In this review, the performance of electrochemical biosensors utilizing immobilized cholinesterases is considered to show their advantages and drawbacks in the determination of anticholinesterase drugs. In addition, common drugs applied in treating neurodegenerative diseases are briefly characterized. The immobilization of enzymes, nature of the signal recorded and its dependence on the transducer modification are considered and the analytical characteristics of appropriate biosensors are summarized for donepezil, huperzine A, rivastigmine, eserine and galantamine as common anti-dementia drugs. Finally, the prospects for the application of AChE-based biosensors in clinical practice are discussed.

## 1. Introduction

Acetylcholinesterase (AChE, EC 3.1.1.7) plays a crucial role in the transduction of nerve impulses [1]. It facilitates the hydrolysis of the natural neurotransmitter acetylcholine into choline and acetic acid [2]. This reaction occurs in the synaptic gap and results in the discrete transduction of impulses between axons. AChE has long been utilized in analytical devices designed for detecting compounds that inhibit the reaction, causing severe consequences for human health [3].

Chemical warfare agents (sarin, soman, VX gases) [4,5,6] and organophosphate and carbamate pesticides (malathion, parathion, carbaryl as examples) [7,8,9] are mostly considered as target analytes. Their contact with the enzyme incorporated in the biosensor assembly results in a lower rate of enzymatic reaction and in decay in the quantities of the hydrolysis products detected with appropriate transducers. Although chemical weapons are mostly destructed in accordance with the Chemical Weapons Convention, incidents of their use have been reported in the Iraq and Syria conflicts and in chemical terrorist attacks (sarin application in Tokyo subway by Aum Shinrikyo, 1995 [10]).

Most of the nerve agents mentioned exert an irreversible effect on the AChE activity [11]. This assumes certain requirements of the measurement protocol and biosensor assembling, e.g., the single use of an immobilized enzyme contacted with the inhibitor, its low loading on the sensor interface and a significance of the measurement precision regarding the signal to the substrate. Irreversible inhibition is mostly measured via a two-step protocol, with the incubation stage performed in the absence of the substrate and the following addition of synthetic substrates and the enzyme activity assessment [12].

Recently, the attention of researchers involved in the development of AChE biosensors has been turned to another class of inhibitors that exert a reversible influence on the enzyme activity. These biosensors have been developed for the determination of drugs applied in the treatment of neurodegenerative diseases. From those, Alzheimer’s disease (AD) is one of the most serious concerns [13]. AD is the leading form of dementia in aging people. According to the WHO, the number of people afflicted by AD will reach 66 million in 2030 and about 115 million by 2050. AD causes neuronal cell death, the development of neurofibrillary tangles composed of hyperphosphorylated tau protein and the formation of amyloid beta (Aβ) or senile plaques [14], as well as significant losses of cortically projecting cholinergic neurons and a reduction in the presynaptic markers of the cholinergic system. Low levels of acetylcholine in patients are associated with memory loss and a gradual learning decrease [15]. AD treatment is limited to symptomatic and palliative medications and no drugs have so far been designed to stop the progression of this disease. Thus, the low level of acetylcholine can be compensated by AChE inhibition followed by a decreasing rate of acetylcholine hydrolysis. For this reason, anticholinesterase drugs are recommended for AD treatment. The efficiency and dose vary from patient to patient and are the subject of monitoring and pharmacokinetics study. In addition, searching for reversible inhibitors as potential drugs is demanded in the search for new drug formulations with an extended efficiency and lower by-effects.

Although the AChE sensors developed for irreversible inhibitor detection can be used for reversible inhibition assessment, the requirements for the assembly of the biosensor and measurements protocol differ from each other. The necessity to measure the signal in the presence of both reactants, i.e., inhibitor and substrate, causes the different behavior of such biosensors and requires additional efforts to reach the sensitivity and accuracy of measurement required for a close and timely control of the drug dosage.

Recently, a number of comprehensive reviews devoted to various aspects of the development and application of enzyme biosensors for inhibitor determination [11,12,16] and to anti-AD drugs–cholinesterase interactions [17,18,19] have been published. However, they pay little attention to electrochemical signal transduction, biosensor assembling and their analytical characteristics. In this review, the design of the electrochemical cholinesterase-based biosensors intended for the anti-AD drug determination is for the first time considered step by step from the brief characterization of the drugs and their interactions with enzymes to cholinesterase immobilization, amperometric and potentiometric signals transduction and figures of merit of the drug determination. Finally, the advantages and drawbacks of the biosensors are summarized with particular emphasis on the spiked and real samples’ assay.

## 2. Cholinesterase and Anticholinesterase Agents

### 2.1. Cholinesterases

**Figure 1:**
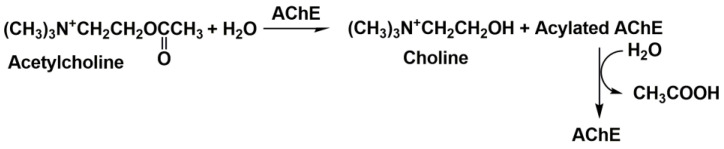
Acetylcholine hydrolysis catalyzed by AChE.

Cholinesterases constitute a group of esterases that hydrolyze choline esters at a higher rate over other esters [17]. They eliminate acetylcholine in cholinergic synapses within several milliseconds after its release, allowing for the temporal control of muscle contraction. Cholinesterases are widely present in biological species. Cholinesterases are classified by the maximal rate of the substrate hydrolysis. Thus, AChE (EC 3.1.1.7) exerts the highest rate of acetylcholine hydrolysis and butyrylcholinesterase (BChE, EC 3.1.1.8) of butyrylcholine. Both enzymes are most frequently mentioned in the inhibitor assay. AChE is mainly bonded to the cellular membranes of excitable tissues and erythrocytes and hydrolyzes acetylcholine (Figure 1) to terminate the transmission of the neural impulse in the presynaptic cell to the somatic neuromuscular junction. Most of the drugs applied in AD treatment inhibit AChE.

BChE, a serum cholinesterase, is found in plasma, liver and muscle tissues. The biochemical function of BChE is probably related to scavenging biogenic species. BChE participates in the biochemical conversion of some drugs [20]. Thus, aspirin is hydrolyzed by BChE to salicylic acid and acetic acid [21]. Plasma BChE also hydrolyzes the prodrugs of antiviral agents, e.g., oseltamivir phosphate (Tamiflu) [22] and acyclovir esters [23]. Irinotecan, an anticancer drug approved for colon cancer treatment, forms an active metabolite, SN-38 [24]. A high-dose treatment produces symptoms of cholinergic toxicity due to the AChE inhibition [25]. Fast BChE-aided hydrolysis of the (+)-cocaine isomer explains its low pharmacological activity. Meanwhile, the (−)-cocaine isomer has a plasma half-life of 45–60 min, sufficient for central nervous system effects [26]. Heroin (pro-morphine) is deacetylated to 6-acetylmorphine and morphine is able to bind to the opioid receptor and suppress pain [27]. 6-Acetylmorphine formed in the presence of plasma BChE easily enters the brain.

The structure of AChE and BChE is well known. Both enzymes contain a characteristic hydrophobic gorge intruded into the surface of the protein globule, where the acetylcholine cleavage occurs. In AChE, binding the substrate is performed by two phenylalanine residues, which sees aromatic rings protrude into the gorge [28]. The acetylcholine hydrolysis takes place at the esteratic site comprised of the catalytic triad serine–histidine–glutamine. So-called anionic and esteratic centers are different in their affinity toward acetylcholine and choline. Rather small conformational changes in the enzyme during the substrate binding explain the high specific activity of the enzyme. The AChE molecule catalyzes the break-down of about 10,000 acetylcholine molecules per second.

Assuming the steady-state kinetics of all the stages of the substrate conversion (Equation (1)), the reaction rate ν corresponds to the Michaelis–Menten equation (Equation (2)). Firstly, the cholinesterase *E* forms a reversible molecular complex *E-S* with the substrate *S*. Then, it is converted to so-called acylated cholinesterase *E-A* by the choline (HX) release. The reaction is finished by a fast *E-A* hydrolysis and recovery of the initial enzyme molecule. Here, in the description, *k_i_* are the rate constants of the individual reaction steps, *k_cat_* is the catalytic constant (turnover number), *K_M_* is the Michaelis constant and *V_max_* is a maximal rate of the enzymatic reaction.



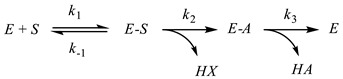

(1)



(2) $$\nu =\frac{V_{\max}c_{S}}{K_{M}+c_{S}}=\frac{k_{cat}c_{E}^{t}c_{S}}{K_{M}+c_{S}};k_{cat}=\frac{k_{2}k_{3}}{k_{2}+k_{3}};K_{M}=\frac{k_{3}(k_{-1}+k_{2})}{k_{2}+k_{3}}$$

**Figure 2:**
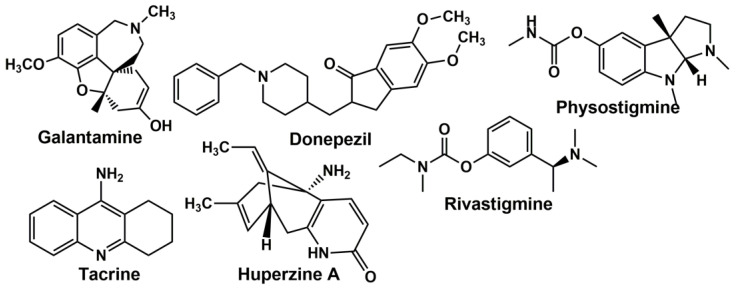
AChE inhibitors used for neurodegenerative diseases treatment.

For AChE from human erythrocytes, the *K_M_* value for acetylcholine and acetylthiocholine is equal to 0.09 (*k_cat_* = 7 × 10^5^ min^−1^) and 0.057 mM, respectively [29]. Physostigmine, an alkaloid extracted from *Physostigma venosum*, was the first known anticholinesterase agent that found medicinal application [30]. Clinical application of cholinesterase inhibitors for AD treatment started in the 1980s [31]. To date, 4 inhibitors have been approved by the Food and Drug Administration (FDA) for AD treatment and more than 20 in various stages of assessment. In accordance with their chemical nature, they can be classified as piperidine derivatives (donepezil), carbamates (rivastigmine, physostigmine, eptastigmine), alkaloids (galantamine, huperzine A), acridine (tacrine) and organophosphates (metrifonate, no longer used in clinical development). The structures of cholinesterase inhibitors applied for AD treatment are presented in Figure 2.

In a kinetic sense, anti-AD inhibitors are reversible because they interact with the enzyme in the presence of the substrate and affect the reaction in both directions (formation and hydrolysis of the enzyme–inhibitor complex). Thus, tacrine and donepezil react with cholinesterases near the active site and do not produce a covalent enzyme–inhibitor complex. Carbamate inhibitors form carbamoylated AChE, which is spontaneously hydrolyzed to the active enzyme. Such a type of inhibition is also called pseudo-irreversible inhibition. From the carbamates tested in AD treatment (eptastigmine, quilostigmine, phenserine, tolserine), only physostigmine was approved by the FDA [32].

Reversible inhibitions show the diversity of the kinetics, which depends on the relative reactivity of the enzyme–inhibitor and enzyme–substrate complexes. Many AChE inhibitors contain cationic groups that mimic the trimethylammonium terminal group of acetylcholine. Other reversible inhibitors can interact with the allosteric center placed near the active site of the enzyme. This also reversibly alters the enzyme activity. Interaction with the allosteric center does not fully suppress the substrate conversion.

The kinetic consideration of reversible inhibition is mainly based on some assumptions, e.g., the fulfilment of the Michaelis–Menten kinetics and 1:1 stoichiometry of the enzyme–inhibitor interaction. Some of them allow a rather simple kinetic description of the reaction and coincide well with the experimental data obtained with reactant solutions.

In *competitive inhibition* (Equation (3)), an inhibitor competes for the occupancy of a substrate-binding site on an enzyme globule. The enzyme–substrate complex *E-S* is inaccessible for the inhibitor attack.



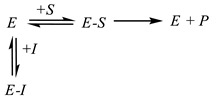

(3)


For competitive inhibition, *V_max_* remains the same as in the absence of an inhibitor and the Michaelis constant *K_M_* decreases linearly with the increasing inhibitor concentration.

*Noncompetitive inhibition* corresponds to the case when an inhibitor binds the site distinct from an enzyme active site. This interaction affects the substrate binding but not the reactivity of the enzyme–substrate complex (Equation (4)).



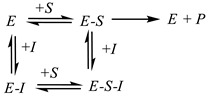

(4)


In this case, the *K_M_* value remains constant, and the *V_max_* decreases with the increasing inhibitor concentration. Pure noncompetitive binding is a most simple example of the allosteric effect when changes in an inhibitor-binding site cause changes in the rate of the substrate conversion.

*Uncompetitive inhibition* assumes the interaction of an inhibitor only with the enzyme–substrate complex. It is rarely observed for cholinesterases. For uncompetitive inhibition, the *V_max_*/*K_M_* ratio does not depend on the inhibitor concentration.

For kinetically pure inhibition types, the conclusion of the mechanism of enzyme–inhibitor interaction follows from the experiments with varying concentrations of a substrate and an inhibitor. In more detail, the kinetic analysis of inhibition is presented in some books and reviews [33,34,35].

Immobilization of an enzyme affects its sensitivity toward an inhibitor. Normally, it becomes lower due to the steric and electrostatic limitations of the substrate–inhibitor transfer to the enzyme location. In addition, the choice of the substrate concentration can be influenced by non-specific adsorption on the enzyme carrier, pH variation in the enzymatic film or by the requirements of reliable signal measurement. Inhibitors exerting a different mechanism of interaction with an enzyme can be compared by the IC_50_ value, i.e., the concentration corresponded to the 50% decay of the enzyme activity. The IC_50_ values can be determined from the signal dependencies, mostly in the logarithmic scale, on the inhibitor concentration, and reflect the relative inhibition ability of the species in similar conditions of the enzyme activity assessment.

The reversible character of inhibition can be empirically established from simple experimental evidence:

- The maximal inhibition remains significantly lower than 100%;
- The addition of a substrate to a solution containing an inhibitor partially restores the signal observed in the absence of an inhibitor;
- The signal toward the inhibitor weakly depends on the contact period (incubation time).

### 2.2. Anticholinesterase Drugs

***Tacrine*** (1,2,3,4-tetrahydroacridin-9-amine) was the first drug that received FDA approval for AD treatment (1993). The hepatotoxicity of the drug was a serious drawback of its regular application [36]. Tacrine inhibition refers to the noncompetitive type and corresponds to the binding anionic site of the enzyme. Tacrine is a more potent inhibitor of BChE than AChE [37]. In addition to the acetylcholine concentration, tacrine increases the extraneuronal concentration of noradrenaline and dopamine [38]. In addition, tacrine affects the glutamate-induced excitotoxicity, causes the induction of a neuroprotective AChE isoform and a reduction in oxidative stress-induced effects [39].

***Rivastigmine*** ((S)-*N*-ethyl-*N*-methyl-3-[1-(dimethylamino)ethyl]-phenyl carbamate hydrogen-(2R,3R)-tartrate, FDA approved in 2000) shows a prolonged noncompetitive inhibitory action both in vivo and in vitro against other carbamate anti-AD drugs and preferentially influences the AChE of the hippocampus and cortex [40]. It binds in the esteratic site of the enzyme and shows equal potency toward AChE and BChE (IC_50_ 48 and 54 μM, respectively, elimination half-life 0.6–2 h) [41]. Major side effects of rivastigmine include stomach pain, weight loss, diarrhea, nausea and vomiting [42]. An overdose of rivastigmine may cause irregular breathing and chest pain.

***Galantamine*** ((4aS,6R,8aS)-5,6,9,10,11,12-hexahydro-3-methoxy-11-methyl-4aH-[1] benzofuro [3a,3,2-ef][2]benzazepin-6-ol, FDA approved in 2001) was used first in Eastern Europe and Russia for the treatment of myopathy, myasthenia and sensory deficits associated with the central neural system [43]. It acts as a competitive AChE inhibitor and allosteric modulator of nicotinic acetylcholine receptors in the brain. This modulation improves the AChE release from presynaptic terminations [44]. Galantamine has a more than 10-fold selectivity to AChE against BChE (IC_50_ 0.8 vs. 7.4 μM). A gradual increase in the galantamine dosage may increase the tolerability of this drug. The recommended daily dosage is 16–24 mg (elimination half-life 5–7 h). The main side effects include convulsions, severe nausea, stomach cramps, vomiting, irregular breathing, confusion, muscle weakness and watering eyes [45].

***Huperzine A*** ((1R,9S,13E)-1-amino-13-ethylidene-11-methyl-6-azatricyclo [7.3.1.02,7] trideca-2(7),3,10-trien-5-one) is a sesquiterpene alkaloid isolated from a club moss *Huperzia serrata*. Huperzine A, a selective potent inhibitor of AChE, is more effective in AD treatment than rivastigmine, galantamine and tacrine [46]. It interacts with the anionic sites of the enzyme via π-π stacking and CH/π-interactions. However, huperzine A may cause mild cholinergic side effects such as nausea, vomiting and diarrhea [47].

***Physostigmine*** ([(3aR,8bS)-3,4,8b-trimethyl-2,3a- dihydro-1H-pyrrolo [2,3-b]indol-7-yl] N-methylcarbamate), also called ***eserine***, is a tertiary amine that reversibly inhibits AChE. Although it was one of the first inhibitors reported in AD treatment, its beneficial effect in a subset of AD patients is considered nowadays as rather small [48].

***Donepezil*** (2-[(1-benzyl-4-piperidyl)methyl]-5,6-dimethoxy-2,3-dihydroinden-1-one) received FDA approval for the treatment of cognitive symptoms in patients with AD in 1996. It shows a high selectivity of AChE binding via the anionic site of the enzyme against BChE ((IC_50_ 22 nM vs. 4.15 μM) [49,50]. The elimination half-life of donepezil via the liver and kidney is assessed as 50–70 h for a single dose per day. In addition, donepezil indirectly stimulates nicotinic and muscarinic receptors via the increased level of acetylcholine in the synapses of the central neural system. Patients treated with high doses of donepezil can suffer from low blood pressure, vomiting, muscle weakness, breathing problems and bradycardia.

***Berberine*** (9,10-dimethoxy-7,8,13,13a-tetradehydro-2′H-[1,3]dioxolo [4′,5′:2,3]berbin-7-ium) is an alkaloid applied in traditional Chinese medicine due to its anti-inflammatory, antimicrobial and antiarrhythmic effects. It is not approved for the administration of neurodegenerative diseases but was actively tested due to a well-pronounced anticholinesterase influence and the ability to suppress many side effects mentioned for other anti-AD drugs [51].

Common anti-AD drugs have been introduced in clinical trials since about 20 years ago. Modern approaches involve the further modification of their structure directed to a higher efficiency in binding AChE and lowering the side effects. A summary of current directions in the design of drugs with the AChE inhibitory action can be found in a review [52].

They include the use of hybrid inhibitors with dual binding sites that are based on traditional building blocks. Thus, tacrine covalently modified with donepezil, hydroxyquinoline, ferulic acid and some primary amines has been described. Some of the hybrid structures utilize the units with antioxidant and neuroprotective functions. It should be noted that AChE inhibitors do not completely stop AD progression. Therefore, there is a need for the further screening of multi-functional drugs able to target AD symptoms including decreased acetylcholine levels, protein misfolding and associated Aβ aggregation, hyperphosphorylation of the tau protein and oxidative stress.

According to the structure–activity studies, novel potent multi-target inhibitors will probably contain a positively charged nitrogen atom linked to a short alkyl chain and separated from oxygen-containing groups (hydroxyl or methoxy) by a two-carbon bridge. Examples of calculations of multi-target directed anticholinesterase drags can be found in [53,54,55].

## 3. Electrochemical AChE Biosensor Design

**Figure 3:**
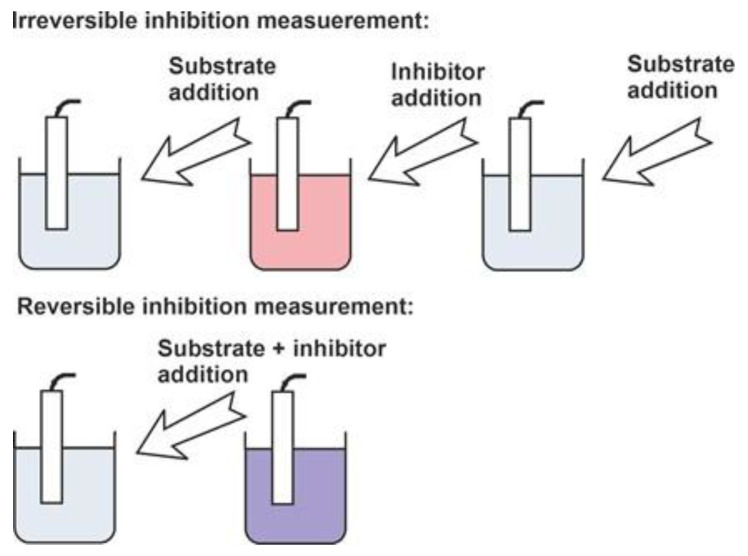
Measurement protocol of irreversible and reversible inhibitor determination with enzyme biosensor.

As was mentioned above, the assessment of cholinesterase inhibitors includes the comparison of the biosensor signals recorded prior to and after the contact of the enzyme with an inhibitor (Figure 3). This makes it possible to calculate the inhibition degree as the relative shift of the enzyme activity and as a measure of the inhibitor content. Even in the case of the direct signal measurement performed in the presence of both the substrate and inhibitor, it is mostly assumed that the initial enzyme activity (for zero inhibitor concentration) is constant and reproducible in the series of experiments required for calibration graph plotting.

For this reason, the stability of the enzyme in the biosensor assembly is a critical factor influencing the inhibition quantification and metrological assessment of the inhibition concentration. It should be noted that enzyme immobilization alters the kinetic parameters of the substrate conversion and can affect the inhibition capability of an analyte. Thus, this calls for the further optimization of the signal measurement conditions even though they were preliminary established for the free enzyme.

### 3.1. Cholinesterase Immobilization

Immobilization of cholinesterase, i.e., its stabilization in an insoluble state on a solid support, preferably on the signal transducer or localized on the transducer interface, is an indispensable part of the biosensor design. The immobilization protocol is intended to establish a long storage and operation period of a biosensor. Meanwhile, its sensitivity toward an inhibitor should be high enough for the analytical applications of the biosensor. This means enzyme manipulations suggested leave the free accessibility of the enzyme active site for the substrate–inhibitor, mostly due to rather low changes in the configuration and flexibility of a protein globule.

**Figure 4:**
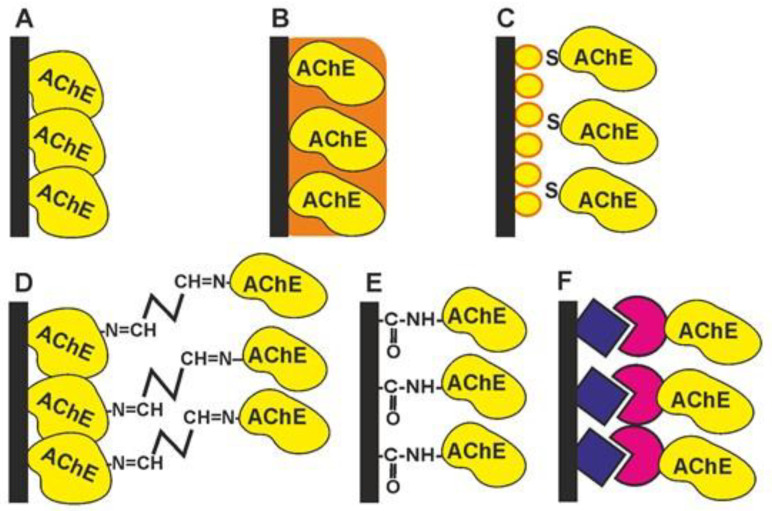
AChE immobilization protocols: (**A**)—physical adsorption; (**B**)—implementation in the polymer film; (**C**)—covalent binding via Au-S bonds; (**D**)—cross-linking with glutaraldehyde; (**E**)—carbodiimide binding to the carboxylated carrier; (**F**)—affinity immobilization via natural receptors.

The following protocols have been described for AChE/BChE immobilization. Most of them were first described for pesticide detection but have also found application for a reversible inhibitors assay. The immobilization schemes are outlined in Figure 4.

- *Physical adsorption on solid support* (Figure 4A). The bare and modified surface of the electrode as a primary signal transducer or plastic films attached to such an electrode can be used as enzyme solid supports. This method offers the high stability of the enzyme during the storage of the biosensor due to the hydrophilic microenvironment of the enzyme established in the surface layer. Physical adsorption on the solid support including entrapment in the polymer gels (Figure 4B) or in the polyelectrolyte complexes makes it possible to preserve the native structure of an enzyme globule and its affinity toward inhibitors. Polyurethane [56], polyaniline [57,58,59], polypyrrole [60], polysiloxanes [61], sodium alginate [62], poly(vinyl acetate) photocurable polymer (PVA-SbQ) [63] and bovine serum albumin (BSA) [64] were used for this purpose. Possible leaching (desorption) of the enzyme can be suppressed by additional cover films deposited or attached to the enzyme layer. A similar approach has been described for the simultaneous immobilization of AChE with an auxiliary enzyme, choline oxidase (ChO) [65]. Carbon nanomaterials offer many advantages as enzyme supports due to a high surface to volume ratio, electroconductivity and a high adsorption capacity [66,67].
- *The formation of self-assembled monolayers* (Figure 4C) is specified because of the high importance of this immobilization protocol for biosensors utilizing golden electrodes or nanoparticles in their assembly [68,69,70,71]. The formation of Au-S bonds offers the site-specific surface immobilization of enzyme molecules. The use of thiolated linkers makes it possible to extend the surface layer and increase its accessibility for inhibitors. Au, as a highly conductive material, improves the conditions of electron transduction and enzyme electric “wiring” and is often combined with other modifiers added to increase the specific surface area (carbon nanomaterials, chitosan films, electropolymerized coatings, etc.).
- *Cross-linking with glutaraldehyde* (Figure 4D) increases the average molar mass of the protein so that the enzyme becomes insoluble and deposits on the solid support [64,72]. Glutaraldehyde interacts with amino and thiol functional groups to form Schiff bases. Although the reaction is reversible, the reverse hydrolysis of the product is less probable in the biosensor operation period. The reaction is mostly performed in the presence of the BSA protein protecting the active site of the enzyme from undesired chemical reactions. The reaction is complicated by the partial oligomerization of glutaraldehyde during storage.
- *Covalent carbodiimide binding* (Figure 4E) with carboxylated carriers [73,74]. Carbodiimide, specifically, *N*-(3-dimethylaminopropyl)-*N′*-ethylcarbodiimide chloride (EDC), forms amide bonds between the carboxylic and amino functions and provides the site-specific immobilization of proteins to carboxylated materials, e.g., carbon nanotubes or carbon black. The reaction is performed in mild conditions (at room temperature). The addition of *N*-hydroxysuccinimide (NHS) prevents the hydrolysis of the unstable intermediate and increases the efficiency of the enzyme binding. Carbodiimide binding is easily combined with the use of Au supports covered with monolayers of mercaptopropionic and mercaptoundecanoic acids.
- *Affine immobilization* (Figure 4F) assumes the use of natural receptors like concanavalin A or an avidin–biotin pair [75,76]. This offers mild and oriented immobilization with a high residual enzyme activity and sensitivity toward inhibitors.

Modern approaches elaborated for AChE introduction in electrochemical biosensors for inhibitor determination are summarized in a review [77]. The immobilization protocols include auxiliary reagents introduced with enzymes in the surface layer. They improve the conditions of signal generation, stabilize the enzyme structure or improve the mechanical stability of the surface layer. A variation in the pH and reactant quantities alters the specific enzyme activity and to some extent the sensitivity of the inhibitor determination. It should be noted that the optimization of the immobilization conditions is frequently based on maximizing the signal toward the substrate. This allows for increasing the measurement accuracy but can decrease the inhibition degree of reversible inhibitors showing a maximal influence in different conditions. From a general consideration, the lower enzyme quantities taken for immobilization increase the sensitivity of the signal toward the inhibitor but decrease the accuracy of the inhibition degree determination. Thus, the content of the enzymatic layer is always a compromise between the arguments of the signal value and inhibition detection.

### 3.2. Cholinesterase Biosensor Signal Measurement

**Figure 5:**
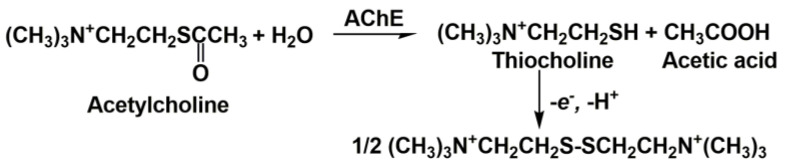
Amperometric sensing of cholinesterase activity with acetylthiocholine as synthetic enzyme substrate.

To date, most cholinesterase biosensors utilize a synthetic substrate, acetylthiocholine, for the monitoring of the enzyme activity and hence for the quantification of the inhibition caused by drugs and other anticholinesterase agents. The reaction is presented in Figure 5.

**Figure 6:**
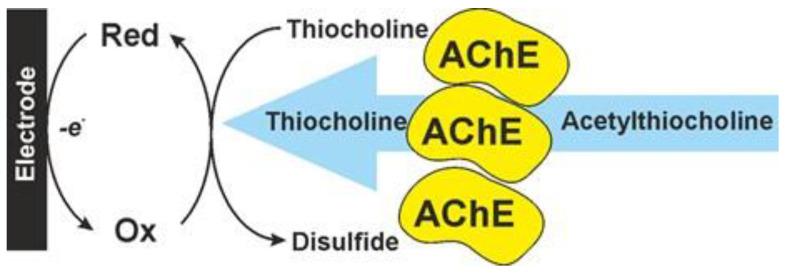
Mediated oxidation of thiocholine in AchE biosensor. Ox and Red are oxidized and reduced forms of a mediator system.

Thiocholine can be directly oxidized to disulfide on carbon electrodes at about 0.6 V. Mediated oxidation is preferred to avoid the passivation of the electrode and to decrease the overpotential of the reaction. For this purpose, Ag [74,78,79,80] and Au nanoparticles [68,69,81,82], Prussian blue [83], Co phthalocyanine [84], 7,7,8,8-tetracyanoquinodimethane (TCNQ) [85], macrocycles bearing hydroquinone units [74,86,87] and metal–organic framework (MOF) particles [88,89] are implemented in the surface layer together with enzyme molecules. Mediators are often combined with each other and carbon nanomaterials. The electron transfer path in mediated AchE biosensors is schematically outlined in Figure 6.

**Figure 7:**
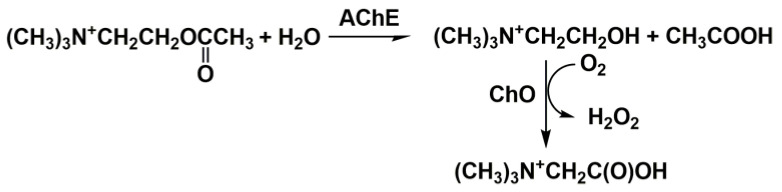
Cholinesterase activity measurement with acetylcholine and two enzymes (AChE and ChO).

The use of the thioanalog of a natural substrate, acetylcholine, complicates the comparison of the inhibition effects with the toxic consequences of poisoning because the nature of the substrate influences the kinetics of reversible inhibition. On the other hand, acetylcholine does not produce an electrochemically active product that could be detected by amperometry. The introduction of a second enzyme, ChO, solves the problem (Figure 7) [65,90].

The design of a bi-enzyme sensor is complicated by the difference in the optimum pH of the AChE and ChO activity and in the specific enzyme activity of the preparations used in the immobilization step. In some cases, one of the enzymes was left in the solution while the second one was immobilized on the electrode interface. The accumulative inhibitory effect of the analytes on the two enzymes increased the sensitivity of the inhibitor determination but was less related to the native influence of the anti-AD drugs on the patients’ health. The signal of the AChE–ChO bi-enzyme sensor can be recorded by mediated oxidation or a reduction of the hydrogen peroxide as a final product of reaction. Au-Pt bimetallic nanoparticles [91], MnO_2_ [92] and phtalocyanines [93] amplify the oxidation of H_2_O_2_ while Prussian blue [94] and CdTe quantum dots [95] mediate its reduction. The latter reaction can also be catalyzed by peroxidase, a third enzyme implemented in the biosensor assembly [96].

The potentiometric measurement of the AChE activity assumes the assessment of the quantities of acetic acid or choline released in the enzymatic reaction (see Figure 1). The simple design of the measurement equipment applied in potentiometry and the open-circuit mode of the signal measurement are the most mentioned advantages of potentiometric biosensors. In the pH measurements, both acetylcholine and acetylthiocholine can be employed as substrates. It should be mentioned that thiocholine formed in the latter case is ionized in basic media. The acidity constant of thiocholine (p*K_a_* = 7.7) is high enough in basic media. Thus, it influences the pH of the solution. This compensates for a lower rate of acetylthiocholine hydrolysis compared to that of acetylcholine. The pH sensitive transducer can be prepared by the deposition of polyaniline, for which the equilibrium potential depends on the pH of the environment. This made it possible to use it as both the enzyme immobilization matrix and signal generation agent [97]. Although the monitoring of the pH changes is one of the oldest approaches in the investigation of enzyme kinetics, it has some obvious drawbacks in the real sample assay and inhibition quantification, i.e., the dependence of the signal on the buffer capacity and pH of the samples tested. The use of choline (thiocholine)-sensitive electrodes based on conventional PVC membranes is an alternative to pH-sensitive biosensors. However, such biosensors were mostly reported for the characterization of the inhibition kinetics [98,99]. The ion-selective membranes consist of the appropriate lipophilic salts of choline and respond on both the substrates (acetylcholine and acetylthiocholine) and the product (choline). The sensitivity of the inhibition measurements was found to be rather modest though the potentiometric sensors were successfully applied for the determination of the inhibition constants. From other potentiometric biosensors, the mediatorless potentiometric detection of H_2_O_2_ can be mentioned for a three-enzyme sensor containing BChE, ChO and peroxidase. It showed a rather high sensitivity in irreversible inhibition measurements [100].

### 3.3. Anti-AD Drugs Determination

As was mentioned above, the design of the cholinesterase-based biosensors developed for drug determination based on their inhibition are mostly derived from appropriate biosensors previously described for pesticide detection. The difference in their application is mostly related to the consequence of reagent addition and data treatment. Nevertheless, some of the biosensors are used for the quantification of enzyme–drug interactions with particular emphasis on the kinetics or sensitivity of the target reactions. Screen-printed Au electrodes combined with a portable potentiostat [101] is one of the examples of such an approach. In this work, human erythrocyte AChE was mixed with the acetylthiocholine and donepezil standard solution in the well of a microtiter plate, thermostated at 37 °C and then spotted on the working electrode. The thiocholine concentration was assessed by differential pulse voltammetry at 0.16 V. The results obtained were compared with a standard Ellman method of colorimetric enzyme assay with 5,5′-dithiobis(2-nitrobenzoic acid) as the chromogenic AChE substrate. The results were expressed as IC_50_ values, which were found to be rather similar to both the electrochemical and colorimetric modes of assay. An S-shaped calibration curve complicated the comparison of the concentration ranges of the drug determination. The method was validated using the AChE containing homogenates from the anterior cortex of mice treated with the donepezil.

A similar approach was reported in [102]. Here, the solutions of AChE and ChO were incubated with acetylthiocholine and drugs (donepezil and tacrine) and the decay of the H_2_O_2_ concentration was monitored in the chronoamperometry mode on the glassy carbon electrode modified with a coordination polymer formed by *L*-cysteine and AgNO_3_. The reaction was first tested for the determination of the cholinesterase activity and H_2_O_2_ concentration and then the IC_50_ of two anti-AD drugs (donepezil and tacrine) was determined in standard solutions.

A microfluidic system based on droplet analysis has been developed for a dose–response inhibition assay [103]. A polydimethylsiloxane chip integrated with microelectrodes has been applied in the gradient generation mode. A whole inhibition assay based on thiocholine oxidation required 6 min. The microfluidic system was tested on carbamate, organophosphate pesticides and tacrine as an anti-AD drug. The latter one exerted inhibition in the concentrations’ range from 0.016 to 87 µM (IC_50_ 37.4 ± 5.8 µM).

In addition to the assessment of relative inhibition abilities, electrochemical biosensors have been described for the determination of the drug concentrations and estimation of possible interferences caused by biological fluid components. It should be noted that the calibration curve of reversible inhibitor determination is not linear and is mostly ascribed by an S-shaped four-parametric logistic function (5) [84,104].

(5) $$I,\%=\frac{A_{1}-A_{2}}{1+{\left(c_{I}/b\right)}^{p}}+A_{2},$$

where $c_{I}$ is an inhibitor concentration, *A*_1_ and *A*_2_ are the upper and lower limits of the curve (ideally equal to zero and 100%, respectively), *b* is a measure of IC_50_ and *p* reflects the sensitivity of the enzyme toward the inhibitor, i.e., the slope of the linear approximation in the middle part of the curve. The parameters of the calibration curve are determined using standard statistical software like those of linear regression but require a higher number of experimental points. A similar approach is used in immunoassay, where the signal depends on the equilibrium of the antigen–antibody interaction. In both cases, linear approximation covers a rather narrow middle part of the whole concentration-dependence event though semi-logarithmic plots (inhibition-log$c_{I}$) are often preferred. The same difference in the metrological assessment of linear and semi-logarithmic plots is the reason for the significant difference in the performance of amperometric and potentiometric sensors used for the assessment of the same inhibitor concentration in similar experimental conditions [97]. Potentiometric sensors are mostly based on the Nernst equation and quantify the pH shift caused by acetic acid released in the substrate hydrolysis. The use of electroactive polymers offers an alternative when similar reactions are monitored by the currents attributed to the shift in the redox equilibrium of the polymeric layer [105].

Flow-through methods of analysis have been gaining increasing attention due to the necessity of screening a large number of samples in similar conditions (biological fluids, drug formulations, waste waters of pharm factories, etc.). In [106], the flow-injection determination of anti-AD drugs has been described with the AChE immobilized on a Au disc. Seven commercially available AChE inhibitors (neostigmine, eserine, tacrine, donepezil, rivastigmine, pyridostigmine and galantamine) were tested and a similarity in the relative inhibition activity was established for various enzyme sources described in the literature. The measurement characteristics were evaluated to obtain both the IC_50_ values and the enzyme–inhibitor equilibrium constants. The flow conditions also made it possible to estimate the dissociation of enzyme–inhibitor complexes and the recovery of the enzyme activity after its contact with an inhibitor.

**Figure 8:**
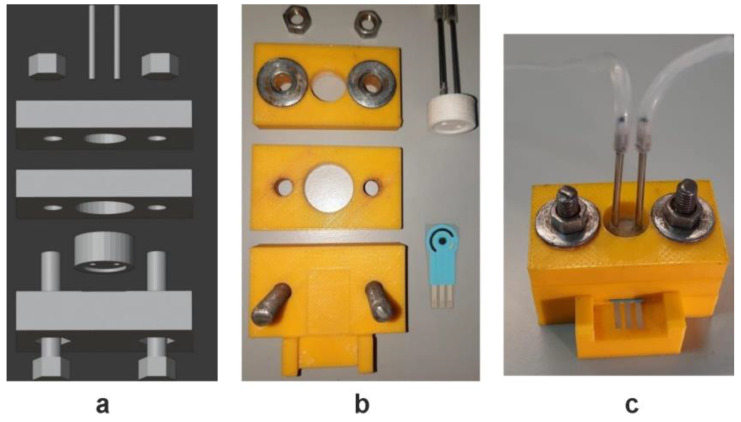
Schematic outline (**a**), disassembled (**b**) and assembled (**c**) flow-through cell with screen-printed electrode and replaceable reactor for AChE inhibitors detection. Adapted from [107], © mdpi open license.

A flow-through replaceable thin film cell with the AChE immobilized to the inner walls via carbodiimide binding has been proposed for the determination of donepezil and berberine [107]. All the disposable parts of the devices were produced by 3D printing from poly(lactic acid). Disassembled and assembled flow-through cells with a screen-printed electrode and replaceable reactor are presented in Figure 8.

The products of the substrate hydrolysis migrated to the screen-printed electrode modified with the macrocyclic derivative of hydroquinone, pillar [5]arene, which was involved in the electron transduction path at a low working potential. The flow-through biosensor device was tested on spiked samples of biological fluids and showed a high recovery of the drug determination.

Some other macrocycles on a pillararene platform have also been tested in the assembly of stationary AChE sensors, alone and together with Ag nanoparticles synthesized in situ by the reduction of AgNO_3_ salts with hydroquinone units of pillararenes [74,80]. Ag accelerates the oxidation of thiocholine and increases the signal of the biosensor toward the substrate due to the accumulation of thiocholine at metal nanoparticles. Meanwhile, the presence of Ag nanoparticles decreases the inhibitor concentrations determined by about one order of magnitude against the same macrocycles taken alone.

Paper-based microfluidic devices are considered as an alternative to flow-through biosensors and intended for the preliminary administration of drugs in neurodegenerative diseases [108]. In those, the enzyme and substrate are located on a different part of the paper support and are combined only on the period of the signal measurement to avoid substrate hydrolysis during the storage period. Such a design, called an origami sensor, assumes the use of screen-printing technologies to obtain conductive pads from carbon containing inks and sample separation parts from commercial membranes that collect the serum and separate red blood cells from their contact with an enzyme. BChE from horse serum was immobilized by spotting on the paper pad and butyrylthiocholine chloride was used as the specific substrate. The thiocholine oxidation was promoted by Prussian blue particles added to the carbon ink. The origami biosensor allowed for assessing the enzyme activity and drugs that inhibit BChE (physostigmine as a model). The measurement period took only 5 min with a deviation of 3.7%.

**Figure 9:**
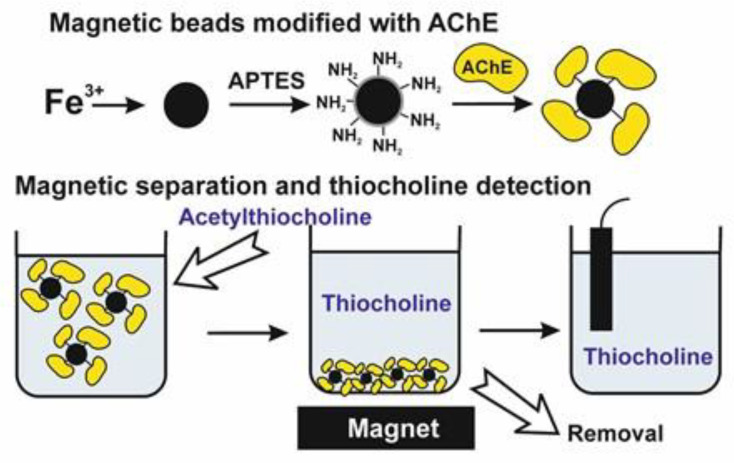
Determination of the AChE activity and anti-DA drugs with magnetic separation of the enzyme and thiocholine formed in acetylthiocholine hydrolysis. Square-wave voltammetry is used for the signal recording. APTES—γ-aminopropyltriethoxysilane.

The idea of the spatial separation of an immobilized enzyme and transducer was also realized in the construction of an electrochemical AChE sensor with aminated magnetic particles used for the immobilization by glutaraldehyde cross-linking [109]. In this work, maghemite superparamagnetic particles were synthesized from the Fe(III) nitrate and modified with the layer containing primary amino groups. After that, the particles were treated with glutaraldehyde and then with the enzyme solution. The reaction resulted in the formation of particles with the active AChE attached to their surface. In the inhibition measurement, the suspension of the particles was first incubated in a microtube with the mixture of an enzyme and inhibitor. After that, the thiocholine formed was separated from the magnetic particles into the microcuvette and the direct oxidation of thiocholine was measured by square-wave voltammetry. The protocol of the biosensor-based measurement is presented in Figure 9.

Galantamine was chosen as a model inhibitor (limit of detection, LOD, of 1.5 µM). The enzyme activity was found to be quite stable in the presence of some organic solvents (alcohols and dimethylsulfoxide). The measurement results showed good correlation with the colorimetric test of the AChE activity (Ellman test).

The analytical performance of the electrochemical cholinesterase biosensors for the determination of the reversible inhibitors applied as the drugs for the administration of neurodegenerative diseases is summarized in Table 1. It should be noted that some of the articles cited contain data on the determination of other traditional AChE inhibitors, preferably organophosphate and carbamate pesticides. They were excluded from consideration. In addition to the results discussed in Section 3.3, some other characteristics of biosensors mentioned above in the description of the biosensor assemblies and enzyme immobilization have been included.

It should be noted that the majority of the sensors presented in the literature are devoted to the determination of appropriate medications in standard solutions. In the case of the inhibition kinetics assessment, the results obtained are compared with the parameters obtained by standard methods used in toxicology and enzymology like the Ellman test of enzyme activity.

Possible interferences present in biological fluids are estimated via the use of spiked samples or of the solutions mimicking the properties of serum plasma or urine (Ringer–Locke solution, artificial urine commercial preparations). On the one hand, they avoid the problems related to the individual diversity of appropriate samples. On another hand, the reliability of such measurements is lower than that based on the reference methods comparison. Indeed, the use of spiked samples with known quantities of drug and artificial biological fluids can be considered as a stepping stone to real sample assays, which is useful and necessary but not fully sufficient for the method validation. Unfortunately, there are no examples of the application of appropriate cholinesterase biosensors for the routine monitoring of drug residues in hospitals.

Regarding point-of-care testing devices, the use of paper-based biosensors in the origami format seems very promising. Its operation is based on intuitively understandable steps that can be performed by unqualified personnel. The immobilization of enzymes provides the stability of their activity within the storage period and a repeatable response toward drugs of interest. It can be expected that such simplified biosensors together with microfluidic devices will find a broader application, especially in developing countries.

## 4. Conclusions

Cholinesterase biosensors offer broad opportunities for the fast and reliable detection of drugs able to reversibly inhibit AChE/BChE activity. Such compounds are demanded in the treatment of neurodegenerative diseases, mainly AD. A growing number of patients who become younger and younger as well as side effects related to the individual sensitivity of patients and difference in the drug metabolism–release increase the risks of drug overdosage and its consequences. Together with the requirements of personalized medicine, this calls for new methods for the preliminary control of such medications and for the selection of new drug candidates intended for cholinesterases binding. Although the analytical application of cholinesterases has been known for more than 60 years, it was mostly related earlier to chemical warfare (sarin, soman, VX gases) and organophosphate and carbamate pesticides (malathion, parathion, carbaryl as examples), called anticholinesterase agents or neurotoxic species. Contrary to those, the determination of reversible inhibitors can be performed in one stage with a simple reactivation of the inhibited enzyme. This simplifies the operation of the AChE biosensors intended for medical use. On another hand, the results (reversible inhibition degree) are more sensitive to the substrate concentration and measurement conditions than in the case of irreversible inhibition. Auxiliary agents present in formulations or in the blood serum of patients can also alter the drug determination. A low selectivity of inhibition detection with enzyme biosensors is a drawback of such an approach. The attempts of the separation of the signal based on the kinetics of enzyme–drug interactions have been performed in homogeneous conditions with the enzyme and analytes present in the same solution with the substrate. If their extension to the biosensor format is successful, the reliability of medications detection will be increased. Together with paper-based and microfluidic formats, this offers good prospects for the application of cholinesterase biosensors for the point-of-care testing of the population.

## Figures and Tables

**Table 1:** Determination of drugs with electrochemical cholinesterase biosensors ^1^.

Surface Layer Assembly	Signal Measurement	Inhibitor, Concentration Range, LOD	Ref.
GCE modified with CB, pillar [6]arene and nanoAg, AChE from electric eel covalently immobilized via carbodiimide binding	Amperometry, mediated thiocholine oxidation	LODs: huperzine A 1.2 nM, galantamine 12.5 nM, donepezil 2.5 nM, berberine 10 nM	[74]
GCE modified with CB and Co phtalocyanine, AChE from electric eel implemented in the PAA-PSS polyelectrolyte complexes	Amperometry, mediated oxidation of thiocholine	Donepezil, 0.1 nM–1.0 µM, IC_50_ 24.7 nM Berberine 10 nM–0.1 mM, IC_50_ 590 nM	[84]
Carbon paste electrode with TTF-TCNQ/ionic liquid, AChE included in the ionic liquid gel	Amperometry, thiocholine oxidation	Eserine 0.1–1000 nM, LOD 0.026 nM	[85]
GCE modified with CB and pillar [6]arene, AChE from electric eel covalently immobilized via carbodiimide binding	Amperometry, mediated thiocholine oxidation	Berberine 10 nM–10 µM, LOD 1.0 nM	[87]
Au electrode modified with PAMAM dendrimer and co-immobilized AChE from electric eel and ChO via Au-S binding (amperometry) or adsorbed on polyaniline (potentiometry)	Amperometry, H_2_O_2_ oxidation, potentiometry, polyaniline equilibrium potential shift	Eserine, LOD 0.1 and 7000 nM for amperometric and potentiometric sensors, respectively	[97]
Screen-printed Au electrode	DPV, thiocholine oxidation, AChE in solution	Donepezil, IC_50_ 28 ± 7 nM	[101]
GCE/rGO covered with *L*-cysteine–Ag(I) coordination polymer in Nafion matrix	Amperometry, H_2_O_2_ reduction in the mixture of AChE–ChO incubated with acetylthiocholine and donepezil	Donepezil, IC_50_ 1.4 nM Tacrine IC_50_ 3.5 nM	[102]
SPCE with poly(methacrylate) and physically adsorbed AChE from electric eel	Amperometry, thiocholine oxidation	Eserine, 3–1000 ng/mL, IC_75_ 2 µg/mL	[104]
Graphite electrode covered with electropolymerized thiophene derivative covalently attached to AChE and ChO	Amperometry	Donepezil, 0.4–5.0 and 5–50 µg/L, LOD 27 ng/L	[105]
Au disk electrode modified with cysteamine and AChE from electric eel covalently attached via Au-S bonding	Amperometry, thiocholine oxidation	IC_50_, µM: donepezil 0.50, neostigmine 0.71, eserine 0.77, tacrine 1.37, galantamine 1.20	[106]
SPCE modified with CB and pillar [5]arene, the AChE from electric eel immobilized via carbodiimide binding to the inner walls of flow cell	Amperometry, mediated thiocholine oxidation	Donepezil 1.0 nM–1.0 µM, IC_50_ 40 nM, LOD 0.5 nM. Berberine 1.0 µM–1.0 mM, IC_50_ 1.24 µM, LOD 0.12 µM	[107]
SPCB on paper support with Prussian blue and BChE from horse serum immobilized via spotting the filter paper	Amperometry, mediated thiocholine oxidation	Physostigmine: 0.01–1.0 µM, LOD 5 nM	[108]
GCE modified with nanoAu/silica gel and physically adsorbed AChE from electric eel	Amperometry, oxidation of thiocholine	Galantamine and neostigmine 0–10 µM	[110]
GCE/nanoPd/g-C_3_N_4_/adsorbed AChE	Amperometry, oxidation of thiocholine	Huperzine A 3.89 nM–20.80 µM, LOD 1.30 nM	[111]
SCE containing α-cyclodextrin in PVC matrix, AChE or BChE in solution incubated with an inhibitor	Potentiometry	Rivastigmine 0.247–1.45, LOD 0.097, Pyridostigmine 0.179–0.975, LOD 0.113, meclofenoxate 375–1725, LOD 30.924, memantine 3.55–18.38, LOD 1.056, methotrexate 1.0–37.0, LOD 3.557, cyclopentolate 2.88–15.6, LOD 0.947, oxfendazole 3.12–34.32, LOD 1.434, carbazepine 2.344–9.50, LOD 0.590 ng/mL	[112]

^1^ Acronyms are presented below in alphabetical order: CB—carbon black; DPV—differential pulse voltammetry; GCE—glassy carbon electrode; LOD—limit of detection; nanoAg—silver nanoparticles; nanoAu—gold nanoparticles; PAA—poly(allylamine hydrochloride); PAMAM—poly(amidoamine) dendrimer; PSS—poly(styrene sulfonate); rGO—reduced graphene oxide; SPCE—screen-printed carbon electrode; TTF-TCNQ—tetrathiafulvalene-tetracyanoquinodimethane.

## Data Availability

Data are contained within the article.
